# Pan-Genome Analysis of Delftia tsuruhatensis Reveals Important Traits Concerning the Genetic Diversity, Pathogenicity, and Biotechnological Properties of the Species

**DOI:** 10.1128/spectrum.02072-21

**Published:** 2022-03-01

**Authors:** Zhiqiu Yin, Xinbei Liu, Chengqian Qian, Li Sun, Shiqi Pang, Jianing Liu, Wei Li, Weiwei Huang, Shiyu Cui, Chengkai Zhang, Weixing Song, Dandan Wang, Zhihong Xie

**Affiliations:** a National Engineering Research Center for Efficient Utilization of Soil and Fertilizer Resources, College of Resources and Environment, Shandong Agricultural University, Tai’an, People’s Republic of China; b Foshan Haitian Flavouring & Food Co. Ltd. (Haitian), Foshan, Guangdong, People’s Republic of China; c School of Biology and Biological Engineering, South China University of Technology, Guangzhou, People’s Republic of China; d College of Plant Protection, Shanxi Agricultural University, Taiyuan, People’s Republic of China; Dublin City University

**Keywords:** *Delftia tsuruhatensis*, genetic diversity, pan-genome, pathogenicity, plant growth promotion

## Abstract

Delftia tsuruhatensis strains have long been known to promote plant growth and biological control. Recently, it has become an emerging opportunistic pathogen in humans. However, the genomic characteristics of the genetic diversity, pathogenicity, and biotechnological properties have not yet been comprehensively investigated. Here, a comparative pan-genome analysis was constructed. The open pan-genome with a large and flexible gene repertoire exhibited a high degree of genetic diversity. The purifying selection was the main force to drive pan-genome evolution. Significant differences were observed in the evolutionary relationship, functional enrichment, and degree of selective pressure between the different components of the pan-genome. A high degree of genetic plasticity was characterized by the determinations of diverse mobile genetic elements (MGEs), massive genomic rearrangement, and horizontal genes. Horizontal gene transfer (HGT) plays an important role in the genetic diversity of this bacterium and the formation of genomic traits. Our results revealed the occurrence of diverse virulence-related elements associated with macromolecular secretion systems, virulence factors associated with multiple nosocomial infections, and antimicrobial resistance, indicating the pathogenic potential. Lateral flagellum, T1SS, T2SS, T6SS, Tad pilus, type IV pilus, and a part of virulence-related genes exhibited general properties, whereas polar flagellum, T4SS, a part of virulence-related genes, and resistance genes presented heterogeneous properties. The pan-genome also harbors abundant genetic traits related to secondary metabolism, carbohydrate active enzymes (CAZymes), and phosphate transporter, indicating rhizosphere adaptation, plant growth promotion, and great potential uses in agriculture and biological control. This study provides comprehensive insights into this uncommon species from the genomic perspective.

**IMPORTANCE** D. tsuruhatensis is considered a plant growth-promoting rhizobacterium (PGPR), an organic pollutant degradation strain, and an emerging opportunistic pathogen to the human. However, the genetic diversity, the evolutionary dynamics, and the genetic basis of these remarkable traits are still little known. We constructed a pan-genome analysis for D. tsuruhatensis and revealed extensive genetic diversity and genetic plasticity exhibited by open pan-genome, diverse mobile genetic elements (MGEs), genomic rearrangement, and horizontal genes. Our results highlight that horizontal gene transfer (HGT) and purifying selection are important forces in D. tsuruhatensis genetic evolution. The abundant virulence-related elements associated with macromolecular secretion systems, virulence factors, and antimicrobial resistance could contribute to the pathogenicity of this bacterium. Therefore, clinical microbiologists need to be aware of D. tsuruhatensis as an opportunistic pathogen. The genetic profiles of secondary metabolism, carbohydrate active enzymes (CAZymes), and phosphate transporter could provide insight into the genetic armory of potential applications for agriculture and biological control of D. tsuruhatensis in general.

## INTRODUCTION

Delftia, a member of the family Comamonadaceae, is a Gram-negative, aerobic, motile, non-spore-forming, and nonfermenting bacterium (1). Delftia tsuruhatensis is a single species in the genus Delftia that was first isolated from sludge in Japan in 2003 (2). It is widespread in rhizosphere soil, activated sludge, and polluted environments. D. tsuruhatensis has a variety of remarkable characteristics. This bacterium has long been investigated as plant growth-promoting rhizobacterium (PGPR) (3–5). PGPRs are a group of rhizosphere bacteria that promote plant growth by a variety of mechanisms, such as the production of auxin, siderophore, and secondary metabolites (6, 7). The utilization of PGPRs could be an important strategy in agricultural production. In early 2005, Han et al. reported a PGPR strain D. tsuruhatensis HR4 isolated from rice rhizosphere, which could effectively suppress the growth of various plant pathogens (4). Subsequently, our colleagues isolated from the tobacco rhizosphere D. tsuruhatensis MTQ3, an environmentally friendly PGPR with the ability to promote antimicrobial activity and plant growth (5, 8). Our previous studies have reported the draft genome sequence and the comparative genomic analysis of MTQ3 and explored functional genes related to antimicrobial activity and environmental adaptation in the genome (5, 8).

D. tsuruhatensis exhibited highly promising metabolic diversity to biodegrade organic pollutants, such as anilines, 2,2-dimethylcyclopropanecarboxamide, terephthalate, protocatechuate, phenols, acetochlor, and chlorobenzene (9–13). Hence, it could be considered a degradation strain for applications with organic pollutants. Furthermore, some researchers reported the antimicrobial activity of D. tsuruhatensis against clinical multidrug-resistant (MDR) pathogens. For instance, Tejman-Yarden et al. reported that the delftibactin A produced by D. tsuruhatensis 2189 exhibited antimicrobial activity against methicillin-resistant Staphylococcus aureus (MRSA), vancomycin resistant Enterococcus (VRE), Acinetobacter baumannii, and Klebsiella pneumoniae (14). Malešević et al. also reported that D. tsuruhatensis11304 inhibited QS systems of P. aeruginosa MDR clinical isolate (15). Hence, the distribution, genetic backgrounds, and relevant synthesis mechanisms of antimicrobial activity and plant growth promotion in D. tsuruhatensis need to be comprehensively studied at the genomic level.

Recently, D. tsuruhatensis has also been found to associate with human infections as an opportunistic pathogen. In 2011, it was first identified as the causative agent of a human catheter-related infection (16). Subsequently, several researchers reported cases of patients with respiratory infections and port-related bacteremia caused by D. tsuruhatensis (17, 18). Furthermore, multidrug-resistant traits in D. tsuruhatensis have also been reported. In early 2007, D. tsuruhatensis A90 was found to carry class 3 integron, which was considered to be associated with resistance genes in pathogens, but no known resistance (19). Recently, two clinical strains, CRS1243 and TR1180, were reported to exhibit resistance to multiple antibiotics and harbor the class 1 integron containing *bla*_IMP-1_ within a Tn*402*-like module and In4-like integron containing multiple resistance genes, respectively (20, 21). However, the underlying mechanisms of pathogenicity and antimicrobial resistance in D. tsuruhatensis species have not yet been comprehensively investigated.

Whole-genome sequencing (WGS) has offered a tremendous advantage for determining the phylogenetic relationship, genetic diversity, virulence-related elements, resistance genes, and biotechnological properties (22, 23). Presently, there are 15 available genomes of D. tsuruhatensis collected by the taxonomically united genome database in EzBioCloud and NCBI GenBank up to September 2021 (24). Pan-genome analysis is a powerful method to explore genomic evolution and genetic diversity of bacterial species. Here, we present a pan-genome analysis of the species D. tsuruhatensis. To expand the understanding of adaptive evolution and divergence in pan-genome, the functional enrichment and selective pressure of core genome and accessory genome were comparatively analyzed. The genomic plasticity and evolution were evaluated by the analysis of mobile genetic elements (MGEs), genomic synteny, and horizontal genes. The key characteristics (e.g., macromolecular secretion systems, genotypic and phenotypic profiles of virulence and resistance genes, secondary metabolite biosynthesis gene clusters, and carbohydrate active enzymes [CAZymes]) that occurred in the pan-genome were investigated to reveal the underlying mechanisms of pathogenicity, antimicrobial resistance, and plant growth promotion in D. tsuruhatensis.

## RESULTS AND DISCUSSION

### Available genomic information for D. tsuruhatensis.

All available sequenced D. tsuruhatensis genomes defined by the taxonomically united genome database in EzBioCloud (24) and NCBI GenBank were collected. The collection contained 15 genomes (Table S1), including two complete genomes (CM13 isolated from murine proximal colonic tissue and TR1180 isolated from the sputum of a 91-year-old female patient with respiratory failure) (21, 25) and two genomes (LMG24775 and LZ-C) originally labeled as Delftia lacustris. These strains were obtained from plant rhizhospheres, water, and host environment, including Homo sapiens, Danio rerio, and murine, exhibiting niche diversity. The genome sizes ranged from 5.737 Mb (MTQ3) to 7.196 Mb (CM13). The genome completeness is greater than 91.6%, and the genome contamination is less than 0.8% (Table S1). The number of rapid annotation using subsystems technology (RAST)-predicted protein-coding genes for the D. tsuruhatensis genomes ranged between 5,227 (MTQ3) and 7,531 (391). The GC content of the D. tsuruhatensis genomes exhibited a minor variation (66.5 ± 0.176%). Table S1 summarizes several key features for the 15 D. tsuruhatensis genomes.

### Whole-genome phylogeny and comparisons of D. tsuruhatensis.

**FIG 1:**
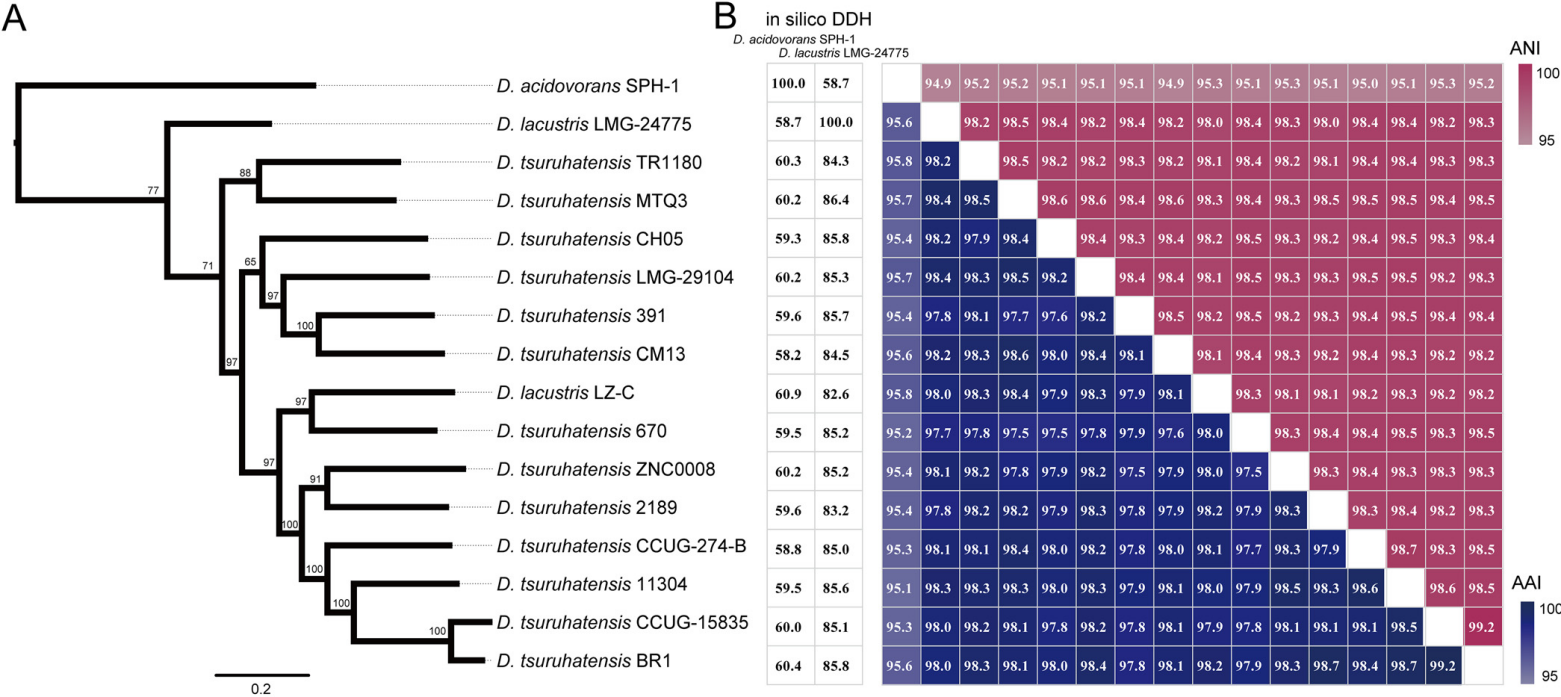
Phylogenetic, *in silico* DNA-DNA hybridization (DDH), whole-genome nucleotide and amino acid identity analysis. (A) Phylogenetic tree based on single-nucleotide polymorphisms (SNPs) across 3,307 single-copy core gene families shared by 15 D. tsuruhatensis genomes and one D. acidovorans genome as an outgroup was constructed by the maximum-likelihood (ML) method with 100 replicates. (B) The values next to the tree indicate *in silico* DDH values. The heat map presents average nucleotide identities (red upper section of the matrix) and amino acid identities (blue lower section of the matrix). ANI, average nucleotide identity.

Whole-genome phylogeny and comparisons have the power to evaluate the evolutionary relationship and phylogenetic position with high resolution. A maximum-likelihood (ML) tree was constructed based on single-nucleotide polymorphisms (SNPs) across 3,307 single-copy core gene families shared by 15 D. tsuruhatensis genomes and 1 Delftia acidovorans genome as an outgroup. The core genome tree appears to have many deep clades and exhibits a relatively low level of clonality (Fig. 1A), suggesting extensive genetic diversity and high recombination rates. Similarly, Vibrio cholerae and Plesiomonas shigelloides have been reported to exhibit low-level clonality because of extensive genetic diversity and high recombination rates (26, 27). We also performed Neighbor-Net network analysis to better visualize the relationships between D. tsuruhatensis genomes. The Neighbor-Net tree (Fig. S1) exhibited a reticular network that is suggestive of recombination.

Whole-genome comparisons were also performed by calculating the average nucleotide identity (ANI), average amino acid identity (AAI), and *in silico* DNA-DNA hybridization (DDH) values, for each genome pair. The ANI and AAI values determined from comparisons between D. tsuruhatensis and D. acidovorans (SPH-1) were 94.9 to 95.3% and 95.1 to 95.8% (Fig. 1B), respectively. The ANI and AAI values shared by D. tsuruhatensis strains were higher than 98.0 and 97.5% (Fig. 1B), respectively. The *in silico* DDH value shared by D. tsuruhatensis and D. acidovorans (SPH-1) was 58.2 to 60.9% (Fig. 1B), which was lower than the 70% species threshold (28). The strain, LMG-24775, originally labeled as D. lacustris, was positioned on the outer branch of D. tsuruhatensis species in the core genome tree. The *in silico* DDH values shared by LMG-24775 and other D. tsuruhatensis strains were 82.6 to 86.4%, which exceeded the recommended 70% threshold value for species circumscription (28). Thus, ANI, AAI, and *in silico* DDH results indicated that LMG-24775 and LZ-C previously labeled as D. lacustris should be corrected to D. tsuruhatensis.

### Pan-genome analyses of D. tsuruhatensis revealed extensive genetic diversity.

**FIG 2:**
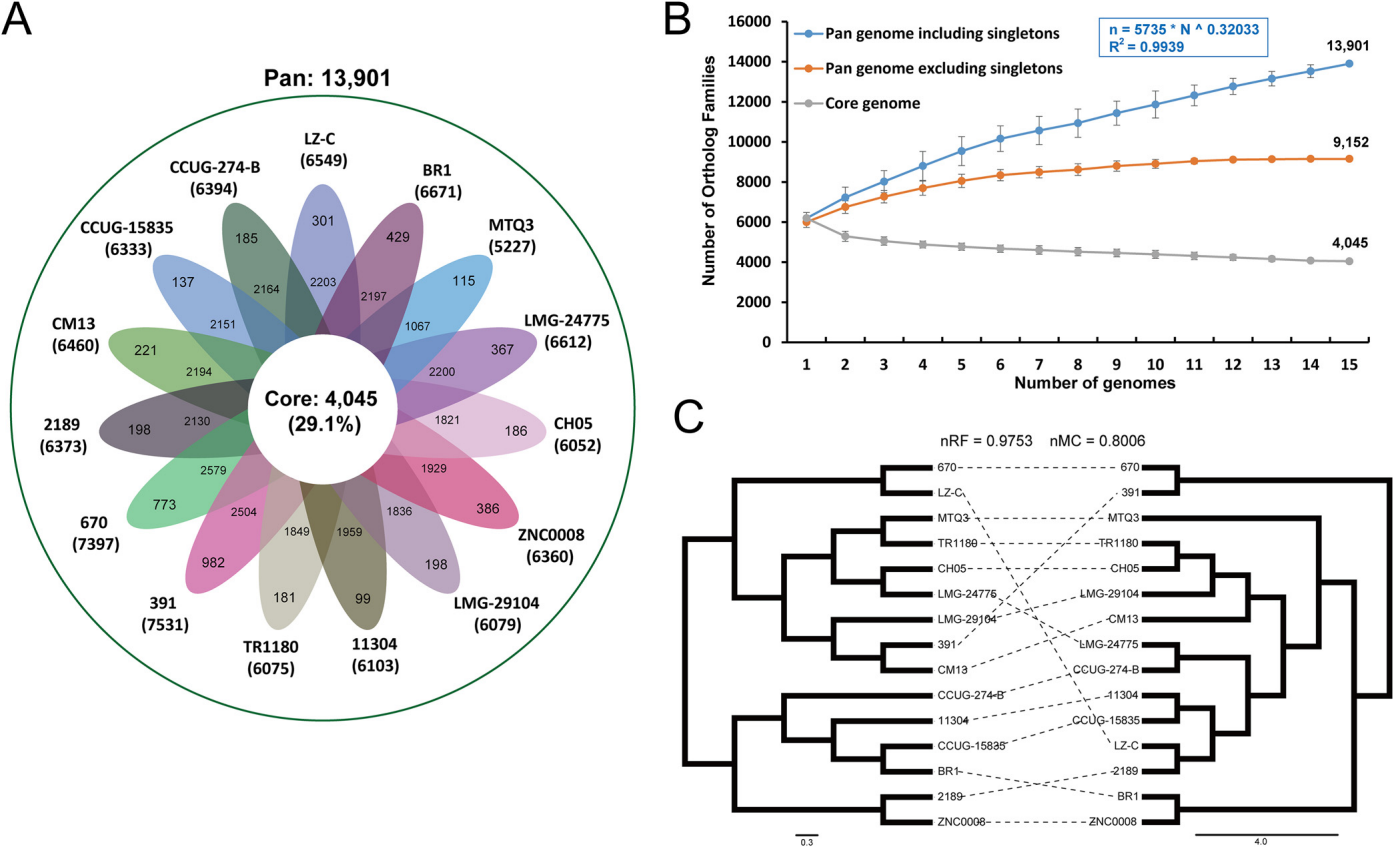
Pan-genome analysis of D. tsuruhatensis. (A) Flower plot of 15 D. tsuruhatensis genomes showing the gene content of core genome (flower center), accessory genome (around the flower center), and strain-specific genes (flower petals). (B) The cumulative curves for the core and pan-genome of D. tsuruhatensis. The curves showed the downward trend of the core gene families and the upward trend of the pan-gene families with the increase in the number of genomes. (C) Comparison of phylogenetic trees generated using single-copy core gene families and pan-genomic distance metric, respectively. Normalized Robinson-Foulds (nRF) and normalized matching-cluster (nMC) scores were used to measure the congruence of the two trees.

To further characterize the genetic diversity of the D. tsuruhatensis species, we estimated the pan-genome represented by 15 D. tsuruhatensis genomes. A total of 13,901 pan-genome gene families were identified (Fig. 2A and Table S2). Among these, 4,045 (29.1%) represented the core genome, and the remaining 9,856 (70.9%) represented the accessory genome (5,098, 36.7%) and strain-specific genes (4,758, 34.2%). The small size of the core genome in D. tsuruhatensis species results in an expansive accessory genome and strain-specific genes. The pan-genome accumulation curve of the increasing number of genomes fits Heaps’ law (*n* = ${\kappa N}^{\gamma }$) pan-genome model (29) (Fig. 2B), with exponent γ = 0.32. A positive exponent (γ > 1) indicated an open pan-genome, suggesting that novel accessory gene families may be identified as additional strains are sampled. Thus, pan-genome analysis indicates that D. tsuruhatensis has a large source gene pool and the potential to adapt to new niches by the acquisition of novel genetic elements. Additionally, it is notable that when singletons (strain-specific) are excluded, we do see a plateau in the pan-genome accumulation curve, suggesting that most undiscovered genes are likely not broadly distributed. Indeed, 4,758 gene families were represented in only one genome, with an average genome containing 317.2 ± 250.9 strain-specific genes, highlighting the high genomic specificity.

To elucidate the importance of accessory genome and strain-specific divergences in shaping genomic architecture, we constructed the core and pan-genome tree of D. tsuruhatensis and compared the phylogenetic topology of the two trees (Fig. 2C). The core genome tree was constructed based on SNPs across 3,441 single-copy core gene families shared by 15 D. tsuruhatensis genomes. The pan-genome tree was constructed based on the presence/absence of pan-gene families in 15 D. tsuruhatensis genomes. The comparison of the two trees could provide a deeper insight into the evolutionary relationship between the core genome and noncore genome. As shown in Fig. 2C, the core and pan-genome tree exhibits the divergence in the topology of the phylogenetic placement and branching order. The relatively discordant topology was also reflected by normalized Robinson-Foulds (nRF = 0.975) and normalized matching-cluster (nMC = 0.801) values. The low congruence in the phylogenetic relationships between the core and pan-genome tree may be due to the occurrence of variable genes, especially the large number of strain-specific genes. These variable gene families might play an important role in the genetic evolution of D. tsuruhatensis and deserve more attention. Our results suggest that genetic diversity has been of great significance in the evolution of D. tsuruhatensis.

### Functional enrichment and pressure selection reveal the divergence between core and pan-genome.

To obtain a deeper understanding of the functional enrichment of each component in the pan-genome, we performed cluster of orthologous group (COG) analysis to categorize the function of pan-gene families. Only 65.6% (9,125 of 13,901) of gene families were assigned to 23 COG functional categories, because a large number of accessory gene families (2,075 of 5,098; 40.7%) and strain-specific genes (2,450 of 4,758; 51.5%) could not be assigned to provisional functions. Functional divergence between the core, accessory, and strain-specific components in pan-genome might reflect the evolutionary dynamic of the D. tsuruhatensis genetic properties. The core genome was significantly enriched in “J: Translation, ribosomal structure and biogenesis” (Fisher’s exact test *P* < 0.01), “T: Signal transduction mechanisms” (Fisher’s exact test *P* = 0.025), “C: Energy production and conversion” (Fisher’s exact test *P* = 0.012), and “H: Coenzyme transport and metabolism” (Fisher’s exact test *P* = 0.033). Both the accessory genome and strain-specific genes were significantly enriched in “L: Replication, recombination and repair” (Fisher’s exact test *P* < 0.01), indicating that recombination was of great significance in evolution. Furthermore, the accessory genome was also mainly responsible for “U: Intracellular trafficking, secretion, and vesicular transport” and “S: Function unknown” (Fisher’s exact test *P* < 0.01), suggesting the acquisition of potential properties associated with host interaction.

**FIG 3:**
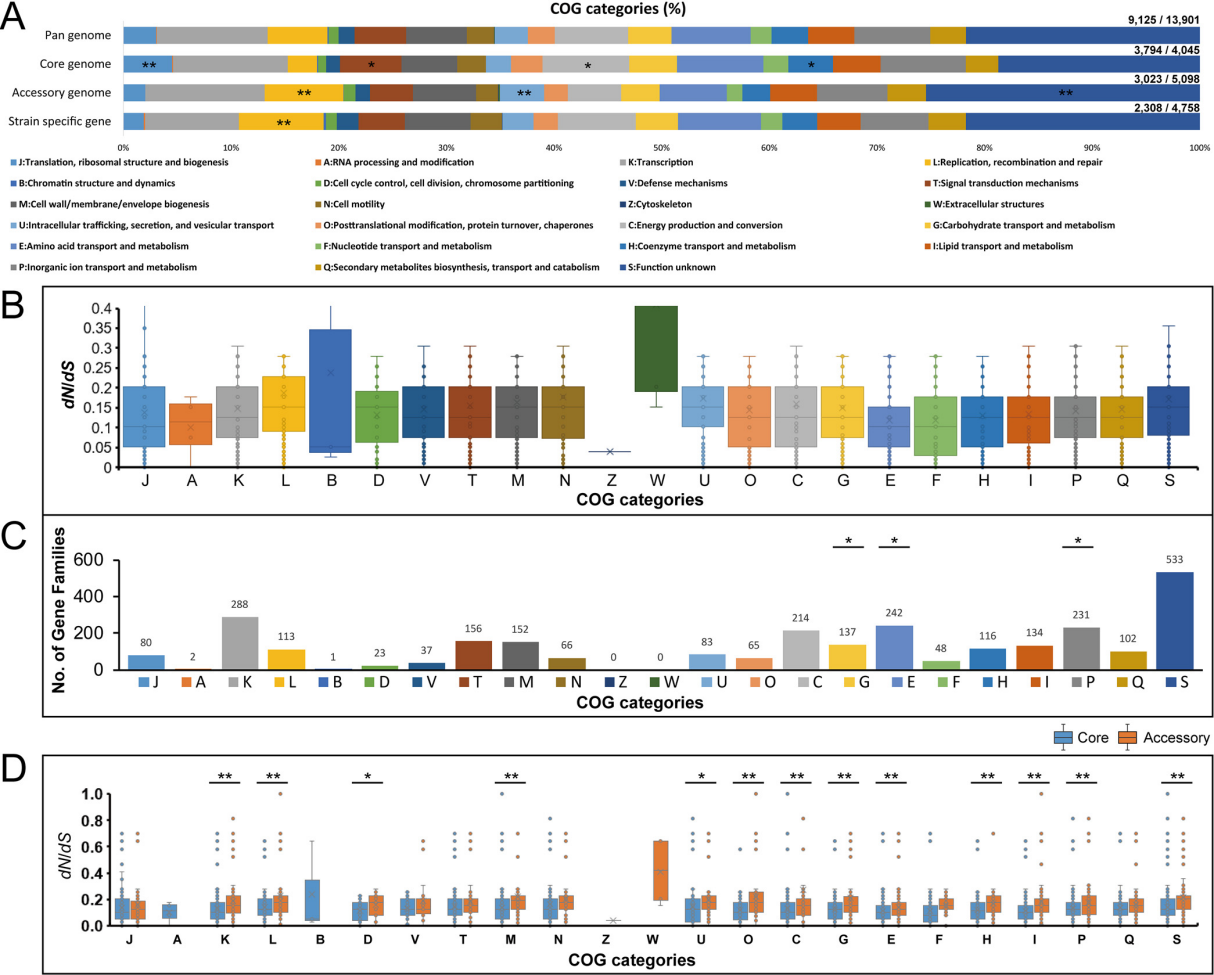
Functional categories and evolutionary dynamics of pan-genome. (A) Distribution of cluster of orthologous group (COG) categories for pan-genome, core genome, accessory genome, and strain-specific genes, respectively. *Fisher’s exact test *P* < 0.05; **Fisher’s exact test *P* < 0.01. (B) Distribution of the nonsynonymous/synonymous rate ratios (*dN*/*dS)* of pan-genome (the gene families shared by less than four strain genomes) in COG functional categories. (C) Distribution of COG categories for gene families with positively selected sites. (D) Comparisons of the *dN*/*dS* values of core genes and accessory genes in COG function categories. *t test *P* < 0.05; **t test *P* < 0.01.

To explore how natural selection shapes the D. tsuruhatensis genetic properties, we performed a codon-level analysis of natural selection on the 6,445 orthologous families (3,982 core gene families and 2,463 accessory gene families) that were present in at least four D. tsuruhatensis strains. The nonsynonymous/synonymous rate ratio (*dN*/*dS*) was calculated to measure the difference in selective pressure. The *dN/dS* values of most of the orthologous families (*N* = 6,360, 98.7%; average *dN*/*dS *= 0.196 ± 0.303) were less than 1, indicating a predominant action of purifying selection in D. tsuruhatensis gene pools. As shown in Fig. 3B, there is divergence in the *dN/dS* values of different functional categories. Our results exhibited the different degrees of purifying selection operating on functional genes, suggesting that the evolutionary strategies may be carried out under different constraints. A total of 85 gene families were identified as positively selected (*dN/dS *> 1), including 6 core gene families and 79 pan-gene families (Table S3). Most of these genes encoded hypothetical proteins, in addition to six genes that encoded fimbrial protein precursor, RelE-like translational repressor toxin, transposase, core protein, 2,3-butanediol dehydrogenase, and cytochrome *b*_561_, respectively. Although entire coding regions were affected by the purifying selection, we identified numerous orthologous families (*N* = 2,906) containing codon sites that were subjected to positive selection (posterior probability ≥ 0.9). Among these, 2,540 orthologous families assigned to COG categories were enriched in “G: Carbohydrate transport and metabolism,” “E: Amino acid transport and metabolism,” and “P: Inorganic ion transport and metabolism” (Fisher’s exact test *P* < 0.05) (Fig. 3C). The presence of positive sites within gene families associated with the transport and metabolism of carbohydrate, amino acid, and inorganic ion might suggest the adaptive evolution of D. tsuruhatensis strains for diverse niches.

We also explored the differences in evolutionary signatures between core and accessory genomes. Overall, the core gene families (average *dN*/*dS *= 0.140 ± 0.148; *t* test, *P < *0.01) were under significantly stronger purifying selection than the accessory gene families (average *dN*/*dS *= 0.287 ± 0.438). Indeed, the housekeeping genes that make up the core genome have a stronger tendency to keep conserving basic functions (30). For instance, the evolutionary constraints of core gene families associated with “Information Storage and Processing” (K: Transcription) and “Cellular Processes and Signaling” (M: Cell wall/membrane/envelope biogenesis) were significantly stronger than those of accessory gene families (*t* test, *P < *0.01) (Fig. 3D). Notably, the core gene families underwent significantly stronger evolutionary constraints in most functional categories of “Metabolism” than accessory gene families (*t* test, *P < *0.01) (Fig. 3D). The functional importance of these core gene families might be the main reason for the stronger purifying selection.

### Genetic plasticity and genomic evolution mediated by numerous mobile genetic elements.

**FIG 4:**
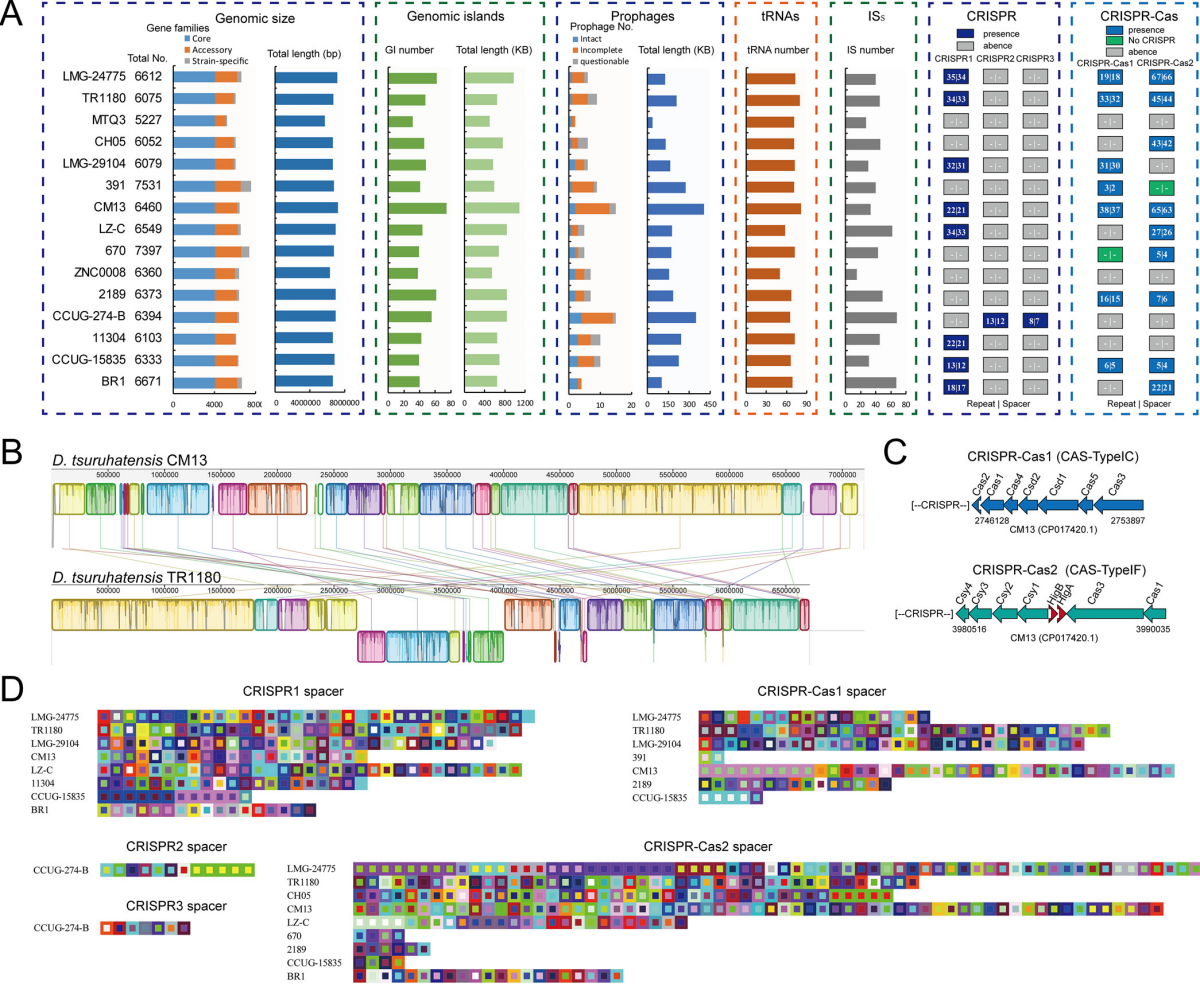
Mobile genetic elements (MGEs) and CRISPR-Cas systems in D. tsuruhatensis. (A) The distribution of MGEs and CRISPR-Cas systems. (B) Genome alignment of two complete genomes: CM13 and TR1180. Synteny blocks are shown as identically colored regions and are linked across the sequences. Regions inverted relative to the CM13 chromosome are shifted downwards from the axis. (C) The genetic organization of CRISPR-Cas systems. (D) The spacer organization of CRISPR. Colored squares represent CRISPR spacers. Spacers of the same color indicate sequence consistency. GI, genomic island.

The genomic size across different D. tsuruhatensis genomes varied from 5.737 Mb (MTQ3) to 7.196 Mb (CM13). The open pan-genome also indicated a high level of genomic diversity. Mobile genetic elements (MGEs) can mediate DNA acquisition and facilitate the expansion of gene pools of bacterial taxa (31). In this study, multiple types of MGEs were identified in the D. tsuruhatensis genomes, including genomic islands (GIs), prophages, and insertion sequences (ISs). These MGEs displayed numerous and heterogeneous distribution models (Fig. 4A). On average, one genome contained 47.1 ± 11.338 GIs (719.8 ± 164.776 kb in size), 7.7 ± 3.693 prophages (196.3 ± 93.142 kb in size), 69.2 ± 7.702 tRNAs, and 42.7 ± 14.844 ISs. GIs and prophages spanned 10.6 ± 1.962% of per genome. The CM13 had the largest genomic size (7.196 Mb) and also the most MGEs, which contained numerous GIs (*n* = 74, 1,084.5 kb in size) and prophages (*n* = 15, 403.5 kb in size), spanning 15.1 and 5.6% of the genome, respectively (Fig. 4A). In contrast, as the smallest genomic size (5.737 Mb), MTQ3 had the least MGEs, which contained GIs (*n* = 31, 497.4 kb in size) and prophage (*n* = 2, 38.4 kb in size), spanning only 8.6 and 0.7% of the genome, respectively. These numerous MGEs in D. tsuruhatensis contributed to its genomic diversity and could be a major driver of horizontal gene transfer (HGT) and strain-specific evolution of D. tsuruhatensis.

Genomic synteny analysis provides evolutionary relationships between genomes. Therefore, we performed the alignment between the two complete genomes, CM13 and TR1180. The alignment exhibited a high level of synteny with rearrangements and inversions (Fig. 4B). A total of 37 synteny blocks was identified, spanning 6,735,844 bp (93.6%) and 6,606,877 bp (98.4%) in CM13 and TR1180, respectively. Among these, 17 synteny blocks represented inversions, spanning 1,354,896 bp (CM13) and 1,335,685 bp (TR1180). Our results suggest that rearrangements and inversions occurred during the genomic evolution of D. tsuruhatensis.

Clustered Repetitively Interspaced Palindromic Repeat (CRISPR) and associated proteins defend microbes against recurrent bacteriophage and plasmid infection (32). Based on the location of the genomes, five distinct CRISPR loci were identified in D. tsuruhatensis (Fig. 4A): three CRISPR loci, CRISPR1 to 3, which are represented as orphan CRISPR locus characterized by the absence of adjacent Cas proteins, and the two remaining CRISPR loci CRISPR-Cas1 and CRISPR-Cas2, which are represented as the CRISPR-Cas system. Most of the genomes carried a CRISPR locus, except for MTQ3 and ZNC0008, which contained fewer MGEs (Fig. 4A). CRISPR1 (8 strains), CRISPR-Cas1 (8 strains), and CRISPR-Cas2 (10 strains) were prevalently present in the D. tsuruhatensis genomes, whereas CRISPR2 and CRISPR3 were only present in CCUG-274-B. The subtypes of CRISPR-Cas1 and 2 were type IC and type IF, respectively. Furthermore, CRISPR-Cas1 in 670 and CRISPR-Cas2 in 391 have no CRISPR. The direct repeat (DR) sequences were highly conserved throughout the locus (Fig. S2). Nevertheless, the spacers exhibited extensive diversity in content and organization. The number of the spacer in CRISPR varied from 2 (CRISPR-Cas1 in 391) to 66 (CRISPR-Cas2 in LMG-24775) (Fig. 4D). Abundant spacers were strain specific. Our results indicated that the strains containing more MGEs might have more than one CRISPR locus with abundant spacers. For instance, CM13 contained CRISPR1 with 21 spacers, CRISPR-Cas1 with 37 spacers, and CRISPR-Cas2 with 63 spacers (Fig. 4A and D), while MTQ3 had no CRISPR. Foreign DNA elements are incorporated into a CRISPR array as spacer sequences (33). Therefore, the diversification of the spacers in the CRISPR loci suggested that extensive gene acquisition occurred in the D. tsuruhatensis genomes.

### Horizontal gene families in D. tsuruhatensis.

Horizontal gene transfer (HGT) is the major driver of the genetic diversity of bacteria (31). The emergence of new phenotypic properties through lateral gene transfer furnishes advantages to adapt diverse niches (34). Here, we examined the potential horizontal genes in the D. tsuruhatensis genomes. We identified 1,573 potential horizontal gene families (11.3% of the pan-genome), of which 662 were core genome, 623 were accessory genome, and 288 were strain-specific genes. Our results indicated that the acquisition of genetic elements by HGT contributed to the open pan-genome of D. tsuruhatensis and the divergence in the genomes of the strains.

**FIG 5:**
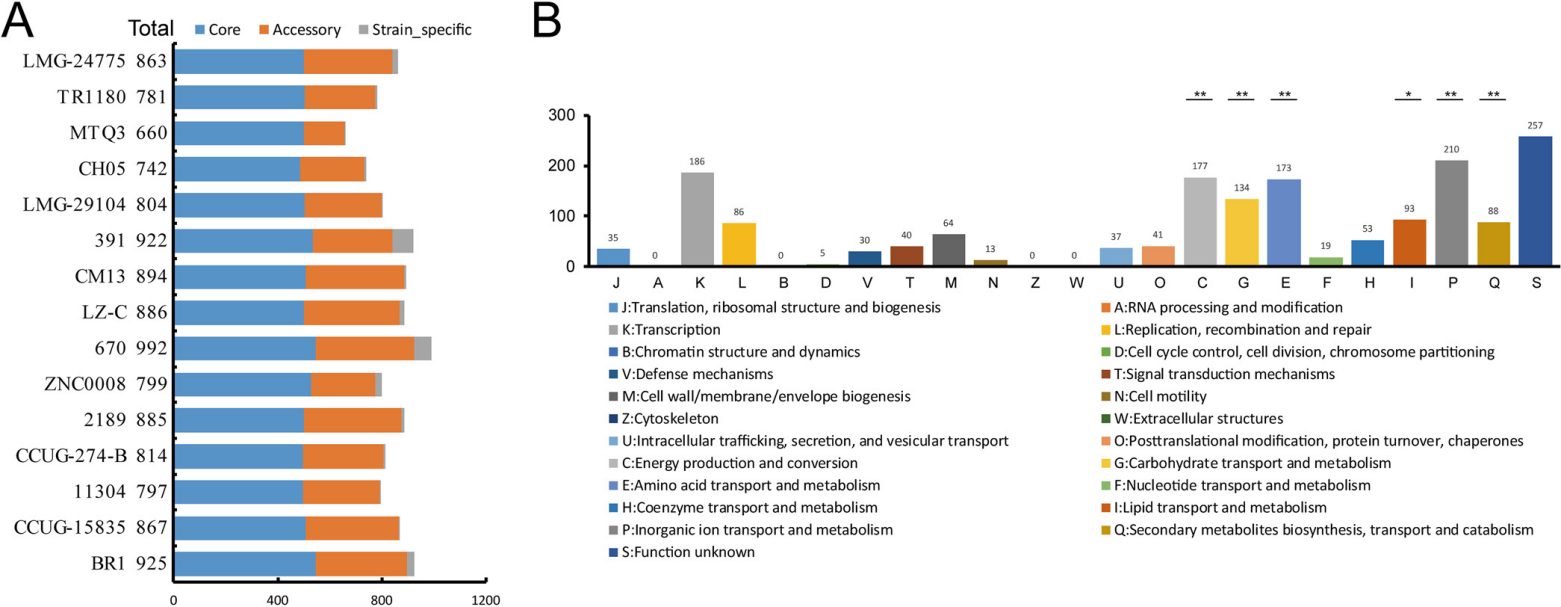
Horizontal gene transfers (HGTs) in D. tsuruhatensis. (A) Distribution of horizontal genes in each strain. (B) Distribution of COG categories for horizontal genes.

An average genome contained 842.1 ± 82.933 horizontal gene families (Fig. 5A). These gene families were significantly involved in the functional categories of “Metabolism,” such as “C: Energy production and conversion,” “G: Carbohydrate transport and metabolism,” “E: Amino acid transport and metabolism,” “P: Inorganic ion transport and metabolism,” “Q: Secondary metabolites biosynthesis, transport and catabolism” (Fisher’s exact test *P* < 0.01), and “I: Lipid transport and metabolism” (Fisher’s exact test *P* = 0.018) (Fig. 5B). It can be inferred that the acquisition of the novel metabolic properties carried by horizontal genes promoted the adaptation of D. tsuruhatensis into diverse niches.

### Macromolecular secretion systems in D. tsuruhatensis, especially for diverse type IV secretion systems.

**FIG 6:**
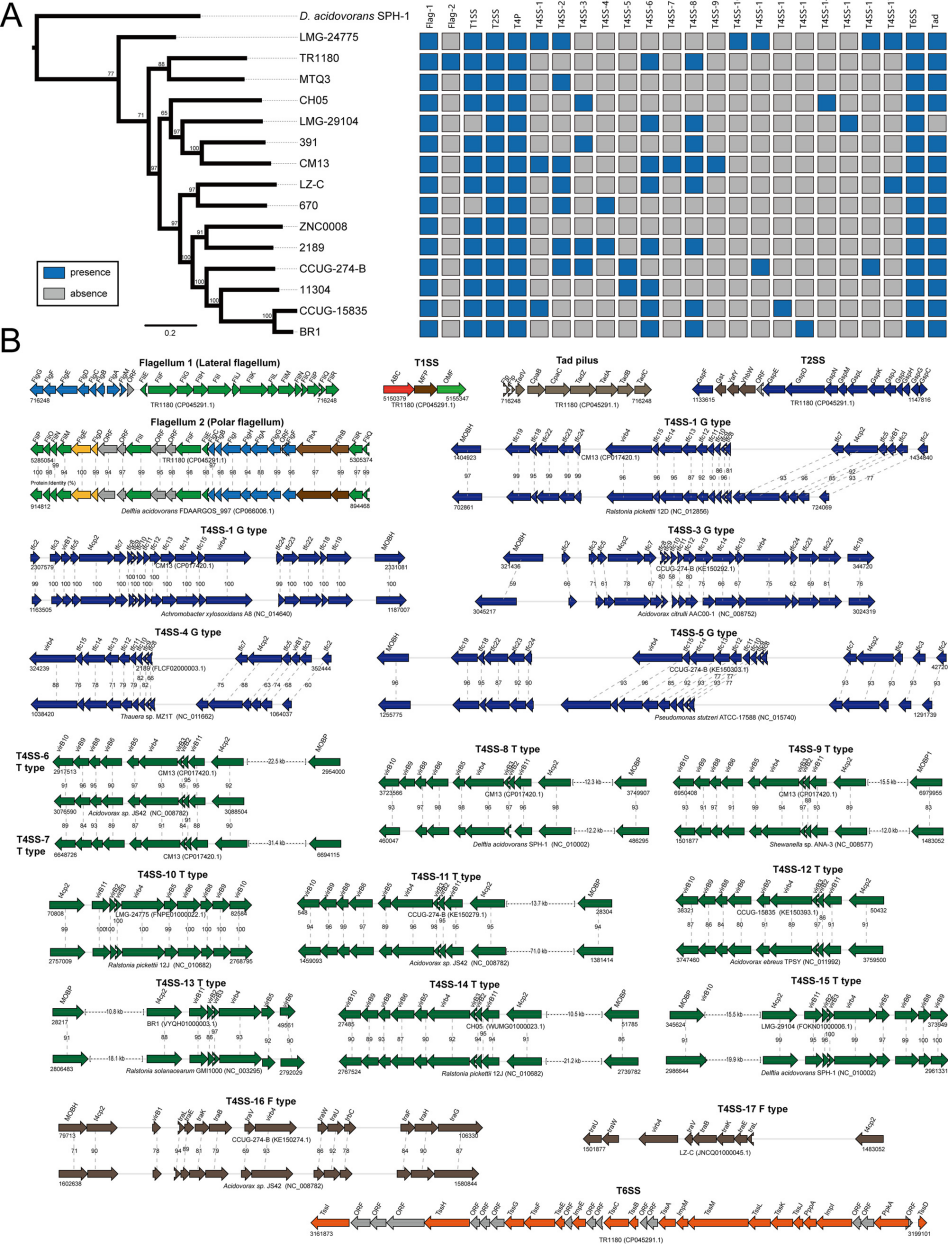
Macromolecular secretion systems in D. tsuruhatensis. (A) Distribution of macromolecular secretion systems. (B) The genetic organization of macromolecular secretion systems. Homologous genes are shown in the same color and linked by dotted lines. The percentages of protein identities of homologous genes are shown.

Macromolecular secretion systems could secrete proteins, DNA, or DNA-protein complex and be involved in key aspects of cell biology, such as nutrient acquisition, host-microbe, or microbe-microbe interactions, motility, environmental adaptation, antibiotic resistance, and pathogenicity (35–37) (Table S4). In this work, we explored the potential gene clusters of macromolecular secretion systems in the D. tsuruhatensis genomes. These gene clusters included those associated with type I (T1SS), II (T2SS), IV (T4SS), VI (T6SS), and IV (T4P); flagellum; and Tad pilus secretion systems. The distributions of the macromolecular secretion systems are represented in Fig. 6A. We identified two distinct gene clusters of flagellum systems: one (designated FLAG-1) that encoded a putative lateral flagellum system and another (designated FLAG-2) that encoded a putative polar flagellum system. FLAG-1 was present in all strains, and FLAG-2 was only present in TR1180. We found that FLAG-2 exhibited the highest homology and almost identical organizations to the polar flagellum locus of D. acidovorans FDAARGOS_997 (Fig. 6B). It can be inferred that the FLAG-2 locus of TR1180 was acquired from closely related species by HGT. Previous studies indicated that lateral flagellum and polar flagellum were associated with virulence in many important tasks such as chemotaxis, adherence, colonization, and invasion (38, 39). Therefore, future studies are required to evaluate the potential role of two flagellum systems in the D. tsuruhatensis pathogenicity, especially for the HGT-derived FLAG-2 locus in the clinical strain TR1180.

The T2SS, T6SS, and T4P loci were distributed in all genomes. The T1SS locus was present in most genomes (13 of 15) in addition to LMG-29104 and 670. The Tad pilus locus was in most genomes (14 of 15) except for LMG-29104. These loci represented a general property of D. tsuruhatensis. T1SS can secrete many proteins, including hemolysins for pathogenesis, some bacteriocins for antibacterial activity, and some extracellular proteases for nutrient acquisition (40). T2SS is implicated in virulence factor secretions and gut colonization (41). T6SS has been reported to contribute to bacterial pathogenesis by the translocation of substrates in the host and competition with other bacteria in their niches (42). Tad pilus is thought to be essential for biofilm formation, pathogenesis, adhesion, or natural transformation in microbes (43). T4P is involved in a variety of functions, including motility, attachment to chemically diverse surfaces, electrical conductance, acquisition of DNA, and secretion of a broad range of structurally distinct protein substrates (44). Therefore, the function of these genetic elements in D. tsuruhatensis and their potential role in pathogenicity deserves attention.

T4SS, as versatile, bacterial membrane-spanning apparatuses, could mediate both genetic exchange and the delivery of effector proteins to target host cells, playing key roles in bacterial genome plasticity and pathogenesis (45). We found 17 distinct gene clusters of T4SS, comprising G (T4SS-1 to -5), T (T4SS-6 to -15), and F (T4SS-16 and -17) types (Fig. 6B). The genetic organization of these T4SSs exhibited extensive diversity. These diverse T4SSs were sporadically distributed in the D. tsuruhatensis genomes, indicating that the presence of T4SSs is strain specific, not a general property. We further performed a BLASTp search of the SecTet4 database to explore the best matches of these T4SS loci. According to the sequence similarity and genetic organization, our results exhibited that the distinct T4SS loci in D. tsuruhatensis showed the highest homology to distinct T6SSs in other species, such as Ralstonia pickettii, Ralstonia solanacearum, Achromobacter xylosoxidans, Acidovorax citrulli, Acidovorax ebreus, Thauera sp., Pseudomonas stutzeri, and Shewanella sp. (Fig. 6B). Both T4SS-6 and -7 exhibited high sequence similarity and identical genetic organization to the T4SS in Acidovorax sp. JS42, indicating twice HGTs or a complex of HGT and duplication occurred in the CM13 genome. Furthermore, we did not find any known T4SS in the SecTet4 database homologous to the T4SS-17. Our analysis revealed that distinct subtypes of T4SS loci in D. tsuruhatensis might be horizontally transferred from diverse donor species. T4SSs have been directly implicated in the horizontal transfer of genes coding for virulence factors, antimicrobial resistance, and other bacterial adaptation traits (46, 47). It can be inferred that the extensively diverse T4SS loci in D. tsuruhatensis might play a key role in genome plasticity and pathogenesis.

### Genotypic and phenotypic profiles of virulence and antimicrobial resistance in D. tsuruhatensis.

**FIG 7:**
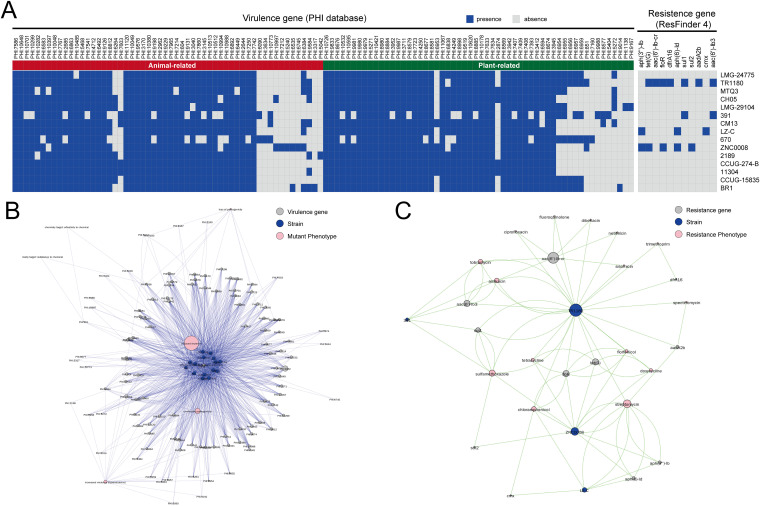
The genotypic and phenotypic profiles of virulence and resistance genes across all 15 D. tsuruhatensis genomes. (A) Heat map of the distribution of virulence and resistance genes. Blue represents the presence of a gene, and gray represents absence. (B) Relationship network for different strains based on virulence genes and mutant phenotypes. (C) Relationship network for different strains based on resistance genes and predicted phenotypes.

We explored the virulence-related genes in D. tsuruhatensis to understand the potential pathogenic mechanisms. A total of 112 gene families were found to match with virulence genes in the PHI-base database (Fig. 7A and Table S5), 80 (71.4%) of which were present in most D. tsuruhatensis genomes (more than 13 strains), the remaining 32 (28.6%) were sporadically distributed in the D. tsuruhatensis genomes. On average, one genome contained 85.2 ± 5.074 potential virulence genes. These virulence genes were predicted to have pathogenicity-related phenotypic outcomes in mutation experiments. The dominantly mutant phenotypes of these genes were “reduced virulence” (*n* = 78, 69.6%) (Fig, 7B), implying that most identified genes were associated with determining virulence. We identified 56 virulence genes that are related to animal hosts, mainly including rodents (*n* = 30), nematodes (*n* = 9), primates (*n* = 6), and moths (*n* = 6). Most virulence genes were associated with determining multiple nosocomial infections, such as urinary tract infection, meningococcal infection, gastric infections, bloodstream infection, skin infection, and prosthetic joint infection (Table S5). Indeed, D. tsuruhatensis as an emerging opportunistic pathogen has been reported to cause respiratory infection, catheter-related infection, and port-related bacteremia (17, 18). Considering the occurrence of a variety of virulence-related genes, clinical microbiologists should consider D. tsuruhatensis, particularly in immunocompromised patients. The remaining 56 virulence genes were associated with plant host and involved in plant disease, mainly including bacterial leaf blight, bacterial wilt, black rot, fire blight, and soft rot (Table S5). Hence, the safety of D. tsuruhatensis as PGPR used in agricultural production still deserves further attention.

Previous studies found that D. tsuruhatensis was susceptible to multiple antibiotics (20, 21). Therefore, we explored the potential profile of resistance genes and phenotypes in D. tsuruhatensis. A total of 11 resistance genes were identified (Fig. 7A). These resistance genes were relevant to resistance to antimicrobials, including aminoglycosides (*aph(3′')-Ib*, *aph(6)-Id aac(6′)-Ib3*, *aac(6′)-Ib-cr*, and *aadA2b*), aminocyclitols (*aadA2b*), fluoroquinolone (*aac(6′)-Ib-cr*), folate pathway antagonist (*sul1*, *sul2*, and *dfrA16*), tetracycline (*tet(G)*), and phenicol (*cmx* and *floR*) (Table S6). These resistance genes were present in four strains: TR1180 (*n* = 7), 391 (*n* = 2), LZ-C (*n* = 3), and ZNC0008 (*n* = 5). Interestingly, strains TR1180 and 391 were isolated from H. sapiens, and ZNC0008 was isolated from D. rerio intestine. We did not detect any resistance genes in other strains. It can be inferred that sporadic HGT might be the major driver of resistance gene acquisition. Indeed, Cheng et al. reported the complete genome of the MDR strain TR1180 and explored In4-like integron associated antimicrobial resistance (21). Meanwhile, class 1 integron and class 3 integron containing several resistance genes were also found in D. tsuruhatensis strains (19, 20). However, we did not find the class 1 integron (accession number KC170993) and class 3 integron (accession number EF469602.1) in the D. tsuruhatensis genomes of this study.

ResFinder 4.0 provides relatively accurate WGS-based prediction of antimicrobial susceptibility (genotype-phenotype concordance of 95% or more) (48, 49). Here, we predicted the phenotypic profile of antimicrobial resistance in D. tsuruhatensis. As shown in Fig. 7C, the D. tsuruhatensis strains were predicted to be resistant to 15 antimicrobials, including aminoglycosides (tobramycin, streptomycin, amikacin, dibekacin, netilmicin, and sisomicin), aminocyclitols (spectinomycin), fluoroquinolone (fluoroquinolone and ciprofloxacin), folate pathway antagonist (sulfamethoxazole and trimethoprim), tetracycline (tetracycline and doxycycline), and phenicol (chloramphenicol and florfenicol) (Table S6). Therefore, the predicted resistance of streptomycin, sulfamethoxazole, and chloramphenicol were distributed across three strains. In general, our findings suggest that clinical microbiologists need to consider D. tsuruhatensis as an emerging pathogen of opportunistic infections with multidrug resistance. The risk of resistance genes linked to MGEs in D. tsuruhatensis deserves further attention.

### Potential growth-promoting traits of D. tsuruhatensis.

**FIG 8:**
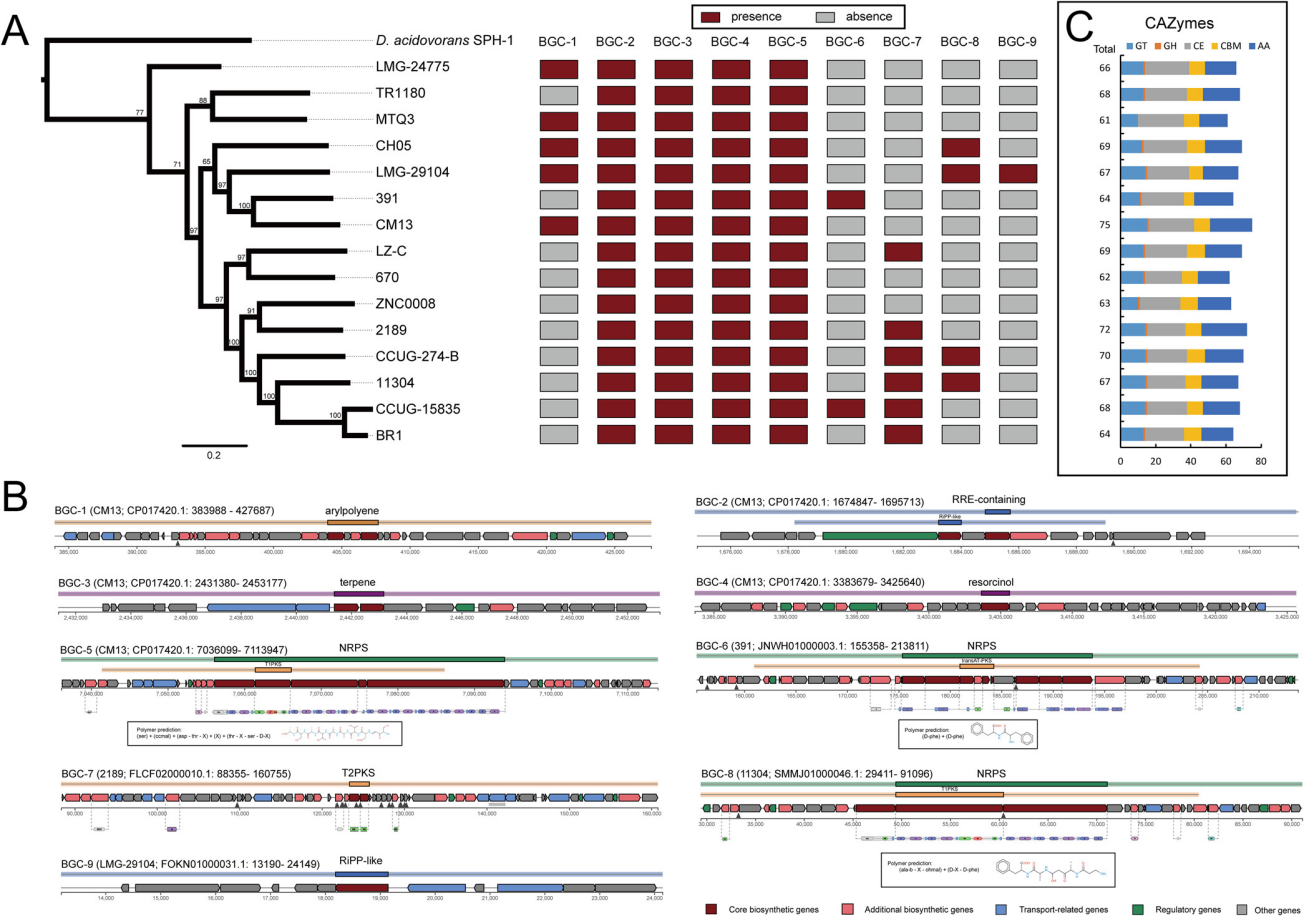
Secondary metabolism elements and carbohydrate active enzymes (CAZymes) in D. tsuruhatensis. (A) Distribution of secondary metabolite elements. (B) Genetic organization of the gene clusters for secondary metabolism. The region in the inset box shows the predicted monomer structures for NRPS-related secondary metabolite elements. (C) Distribution of CAZymes in each strain.

Initially, D. tsuruhatensis have long been known for their ability of plant growth promotion and considered to have important applications in agricultural production (8). Pan-genome analysis identified nine potential biosynthesis gene clusters (BGCs) associated with secondary metabolite synthesis, including aryl polyene (BGC-1), RRE-containing substrate (BGC-2), terpene (BGC-3), resorcinol (BGC-4), NRPS (BGC-5, -6, and -8), T2PKS (BGC-7), and RiPP-like substrate (BGC-9) (Fig. 8). The BGC-2 to -5 were present in all genomes, representing a general property of D. tsuruhatensis. The others were sporadically distributed in the D. tsuruhatensis genomes, indicating that these BGCs were horizontally transferred from other species. Furthermore, we compared the homology of these BGC in antiSMASH database (50). Five BGCs (BGC-1, -5, -6, -7, and -8) were identified that contained the homologous genes from the known BGCs (Fig. S3), but the proportion of homologous genes in most BGCs (except for BGC-5 with 100% homologous genes to the BGC of delftibactin A) was fairly low. BGC-2, -3, -4, and -9 did not show similarities to those present in the antiSMASH database. Previous research has demonstrated the NRP-delftibactin A in Delftia spp. isolates exhibited antimicrobial activity against MDR pathogens like MRSA, VRE, A. baumannii, and K. pneumoniae (14). Therefore, the novel cryptic BGCs in D. tsuruhatensis might represent a treasure trove of novel bioactive compounds with potential biotechnological applications, especially for the two NRPS clusters (BGC-6 and -8). These diverse BGCs might contribute to the promotion of plant growth and biocontrol traits of D. tsuruhatensis.

The complex carbohydrates of plants are the main nutrient sources of rhizosphere microbes. CAZymes are the most important enzymes for complex carbohydrate metabolism. We identified the CAZymes in the D. tsuruhatensis genomes. These genomes contained an abundance of CAZyme-encoding genes for glycosyltransferases (GTs), carbohydrate esterases (CEs), carbohydrate-binding molecules (CBMs), and auxiliary activities (AAs), and a small number of CAZyme-encoding genes for glycoside hydrolases (GHs) (Fig. 8C). On average, one genome contained 67.0 ± 3.817 CAZyme-encoding genes that could encode 12.9 ± 1.506 GTs, 0.9 ± 0.258 GHs, 23.6 ± 1.502 CEs, 9.1 ± 1.033 CBMs, and 20.5 ± 2.532 AAs. Except for 32 CAZyme-encoding gene families in the core genome, these gene families (N = 72) were abundant in accessory and strain-specific genomes (Fig. S4). These diverse CAZymes might enrich the metabolic diversity of D. tsuruhatensis to promote adaptation to diverse environments, particularly in the rhizosphere.

Other characteristics (e.g., indole-3-acetic acid [IAA] production, nitrogen fixation, phosphonate solubilization, and phosphate transporter) associated with plant growth promotion were also investigated. We did not mine genes involved in IAA biosynthesis (indole-3-pyruvic acid [IPyA], indole-3-acetamide [IAM], indole-3-acetonitrile [IAN], and tryptamine pathways [TAM]) (51, 52) and nitrogen fixation (*nif* gene operon composed of *nifBHDKENXhesAnifV*) (53). The *pst* operon (*pstBACS*), encoding a high-affinity, low-velocity, free-phosphate transport system, was found in all D. tsuruhatensis genomes. However, the *phn* operon (*phnA* to *phnQ*), responsible for phosphonate solubilization, was absent (54). Our analysis indicated that D. tsuruhatensis do not seem to have the capability of IAA production, nitrogen fixation, and phosphonate solubilization.

### Conclusions.

In this study, we made a pan-genome analysis with 15 D. tsuruhatensis strains to evaluate the phylogenetic relationship, genetic diversity, genomic plasticity, evolutionary dynamics, pathogenicity, and plant growth-promoting traits of D. tsuruhatensis. Our results provided a comprehensive understanding of D. tsuruhatensis from the genomic perspective. The open pan-genome exhibited extensive genetic diversity with a large and flexible gene repertoire within the accessory genome and strain-specific genes, which promoted rapid evolution. Purifying selection was the main force driving pan-genome evolution. Comparative analysis revealed the significant difference in the evolutionary relationship, functional enrichment, and degree of purifying selection between core genome and noncore genome. D. tsuruhatensis genomes exhibited high levels of genetic plasticity characterized by a large number of MGEs, diverse CRISPRs, genome rearrangements, and horizontal genes, contributing to the expansion of gene pools, especially for genes associated with virulence and resistance.

Our results indicated the occurrence of diverse virulence-related profiles in D. tsuruhatensis, including the macromolecular secretion systems, virulence genes, and resistance genes. In the macromolecular secretion systems, FLAG-1, T1SS, T2SS, T6SS, Tad pilus, and T4P exhibited universal traits, whereas Flg-2 and T4SS were sporadically distributed in D. tsuruhatensis derived by HGTs. In particular, the extensively diverse T4SS loci might play a key role in the genome plasticity and pathogenesis of D. tsuruhatensis. The presence of a variety of virulence genes associated with multiple nosocomial infection underlies the potential pathogenicity strategy of D. tsuruhatensis. Furthermore, we also observed the sporadic occurrence of virulence and resistance genes, indicating that HGT leads to strain-specific pathogenicity and antimicrobial resistance. In general, our findings indicated that D. tsuruhatensis, as an emerging opportunistic pathogen, presented a risk of pathogenicity and antimicrobial resistance.

Our genomic analysis revealed diverse BGCs involved in secondary metabolites in D. tsuruhatensis, highlighting their high potential as PGPRs and biocontrol agents. Moreover, the existence of abundant CAZymes in the accessory and strain-specific genomes reflected the adaptation of diverse environments, particularly in the rhizosphere. The detailed analysis of genome sequence provides useful understanding in the agricultural and biotechnological applications of D. tsuruhatensis.

## MATERIALS AND METHODS

### Genome collection and analysis.

The members of D. tsuruhatensis species were obtained from taxonomically united genome database in EzBioCloud and NCBI GenBank (24). All collected genomes were downloaded from the NCBI GenBank database. The estimates for genome completeness and contamination were performed using CheckM (55). Gene finding and the reannotation of D. tsuruhatensis genomes were performed using the RAST server (56). TheJSpecies 1.2.1 based on MUMmer method (ANIm) (57, 58), CompareM (https://github.com/dparks1134/CompareM), and the online interface of the genome-to-genome distance calculator 2.1 (GGDC) (28) were used to calculate the average nucleotide identity (ANI), amino acid identity (AAI), and *in silico* DNA-DNA hybridization (DDH).

### Pan-genome analysis.

Orthologous groups of protein families of pan-genome were delimited using OrthoFinder2 software with the DIAMOND method (59, 60). The OrthoFinder output files (Orthogroup_Sequences folder) were used to extract pan-genome families (the totality of all genes found across strains), core genome families (genes shared among all strains), accessory genome families (genes shared among more than one strain but not all), and strain-specific genes (genes found only in one strain). Curve fitting of pan-genome was performed using a power-law regression based on Heaps’ law (*n* =${\;\kappa N}^{\gamma }$) (29, 61), where *N* is the number of genomes, κ is a proportionality constant, and the growth exponent *γ* > 0 indicates an open pan-genome. Descriptive statistical analysis was generated using OriginPro 9 software with the Allometric1 model. The gene families of each set were functionally characterized by the COG functional category (62) using eggNOG-mapper software (63).

### Phylogenetic analysis.

The core genome phylogenetic analysis was performed based on SNPs across single-copy core gene families extracted from the OrthoFinder output files. The nucleotide sequences of the single-copy core gene families were extracted according to the protein accession numbers and then aligned using MAFFT software (64). The set of SNPs presented in single-copy core gene families was extracted and then integrated according to the arrangement of the genes on the CM13 genome (complete genome). To avoid phylogenetic confusion, we identified and removed the putative recombinational regions from the SNPs set using ClonalFrameML software (65). The maximum-likelihood (ML) tree was constructed using MEGA 7 (66) with the general time reversible (GTR) model and 100 bootstrap replicates.

The pan-genome tree was constructed based on the gene distribution pattern that D. tsuruhatensis species exhibited, with a binary 1 (presence)/0 (absence) matrix. The Manhattan distance was calculated by the Python NumPy module to measure the evolutionary relationship of strains and then used to construct the neighbor-joining (NJ) tree using MEGA 7 (66). Dendroscope 3 (67) was used to perform the comparison of the core genome tree and pan-genome tree. The congruence between the core genome tree and the pan-genome tree was evaluated by calculating normalized Robinson-Foulds (nRF) and normalized matching-cluster (nMC) scores using the online interface of TreeCmp (68). The Neighbor-Net network was constructed and visualized with SplitsTree4 (69) with uncorrected p-distance transformation.

### Pressure selection analysis.

Positive selection in coding regions can be estimated by calculating the ratio of the nonsynonymous substitution rate to the synonymous substitution rate (*dN*/*dS*). ParaAT software was used to codon-based align the orthologous genes (70), and the Fast Unconstrained Bayesian Approximation (FUBAR) pipeline (71) of HYPHY software was used to measure the *dN*/*dS* ratio at each site in each orthologous gene family.

### Comparative genomic analysis.

The prophages were predicted using the online interface of PHAge search tool – Enhanced Release (PHASTER) (72). The online interface of IslandViewer 4 (73) (integrating three different methods: SIGI-HMM [74], IslandPath-DIMOB [75], and IslandPick [76]) was utilized to identify the genomic islands. Insertion sequences were predicted using the online interface of ISfinder (77). Syntenic analysis was achieved using the ProgressiveMauve package in Mauve software (78), which identified the locally colinear blocks (LCBs) using default parameters. The clustered regularly interspaced short palindromic repeats (CRISPRs) were predicted using the CRISPR recognition tool (CRT1.2) with default parameters (79). The detection and visualization of Macromolecular systems in D. tsuruhatensis *species* were performed using the programs MacSyFinder (35) and TXSScan (36) within Galaxy workflow system (https://galaxy.pasteur.fr/) on the default parameters. The T4SS and T6SS were further analyzed using SecReT4 (45) and SecReT6 (80) on the default parameters, respectively, which annotated the components of T4SS and T6SS on the genome sequences and identified the homologous clusters. The gene cluster related to secondary metabolism was identified and analyzed using antiSMASH (50) on the default parameters. The genes encoding carbohydrate-binding and metabolic enzymes were identified using the dbCAN2 database (81) by HMMER search (82).

### Identification of potential horizontal genes.

HGTector (83) was used to identify the potential horizontal genes in D. tsuruhatensis species. The Delftia (rank: genus; taxon ID: 80865) and Comamonadaceae (rank: family; taxon ID: 80864) were set as self-group and close-group, respectively.

### Identification of the genotypic and phenotypic profiles of virulence factors and resistance genes.

To identify the virulence factors, the protein sequences of all genomes were aligned using BLASTp with an E value cutoff of less than 1e−6, identity of more than 60%, and coverage of more than 60% against the data set from the Pathogen Host Interactions database (PHI-base 5.0) (84). The resistance genes and phenotypes were predicted using ResFinder 4.0 software (48). These results were visualized using heat map R packages.
