# Supplementary material for: Pan-Genome Analysis of Delftia tsuruhatensis Reveals Important Traits Concerning the Genetic Diversity, Pathogenicity, and Biotechnological Properties of the Species
Source: Microbiol Spectr. 2022 Mar 1;10(2):e02072-21. doi: 10.1128/spectrum.02072-21 (PMC9045143; doi:10.1128/spectrum.02072-21)
Supplement: SUPPLEMENTAL FILE 2 — Supplemental material. Download SPECTRUM02072-21_Supp_1_seq12.pdf, PDF file, 0.8 MB [file spectrum02072-21_supp_1_seq12.pdf]

SUPPLEMENTAL MATERIAL FIGURE

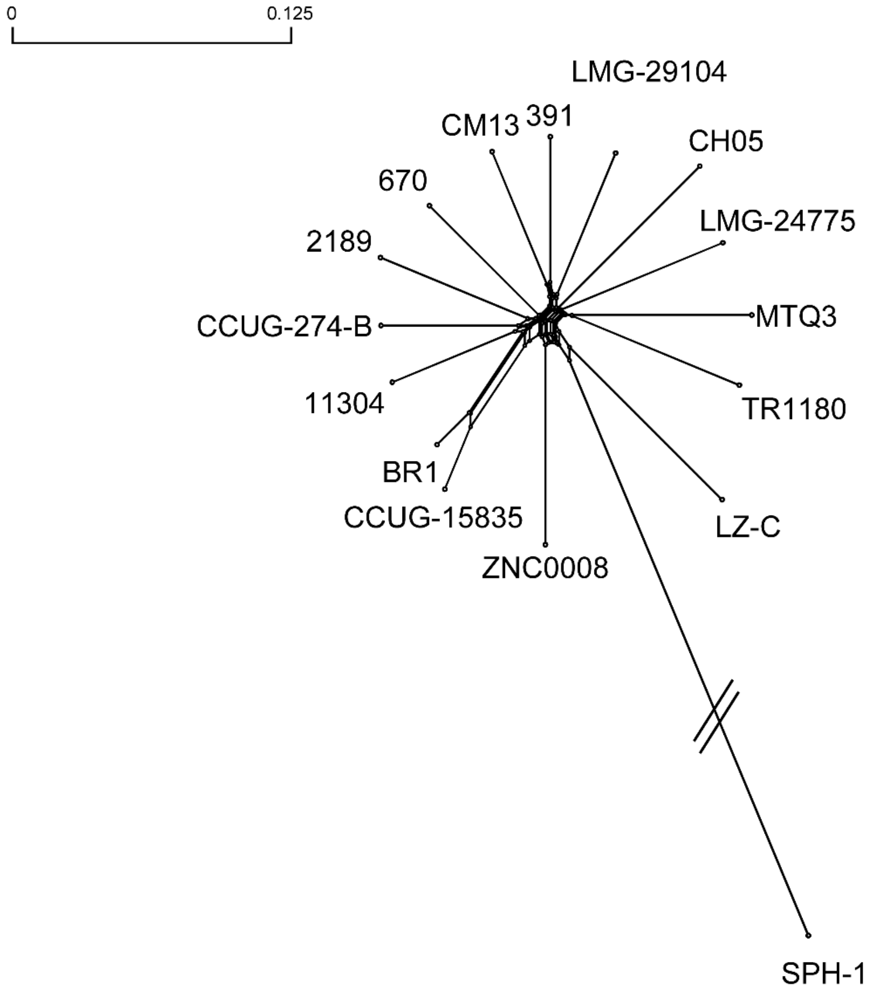

Figure S1. The Neighbor-Net network based on an uncorrected p-distance transformation inferred from the SNPs across 3307 single-copy core gene families shared by 15 *D. tsuruhatensis* genomes and one *D. acidovorans* genome as an outgroup.

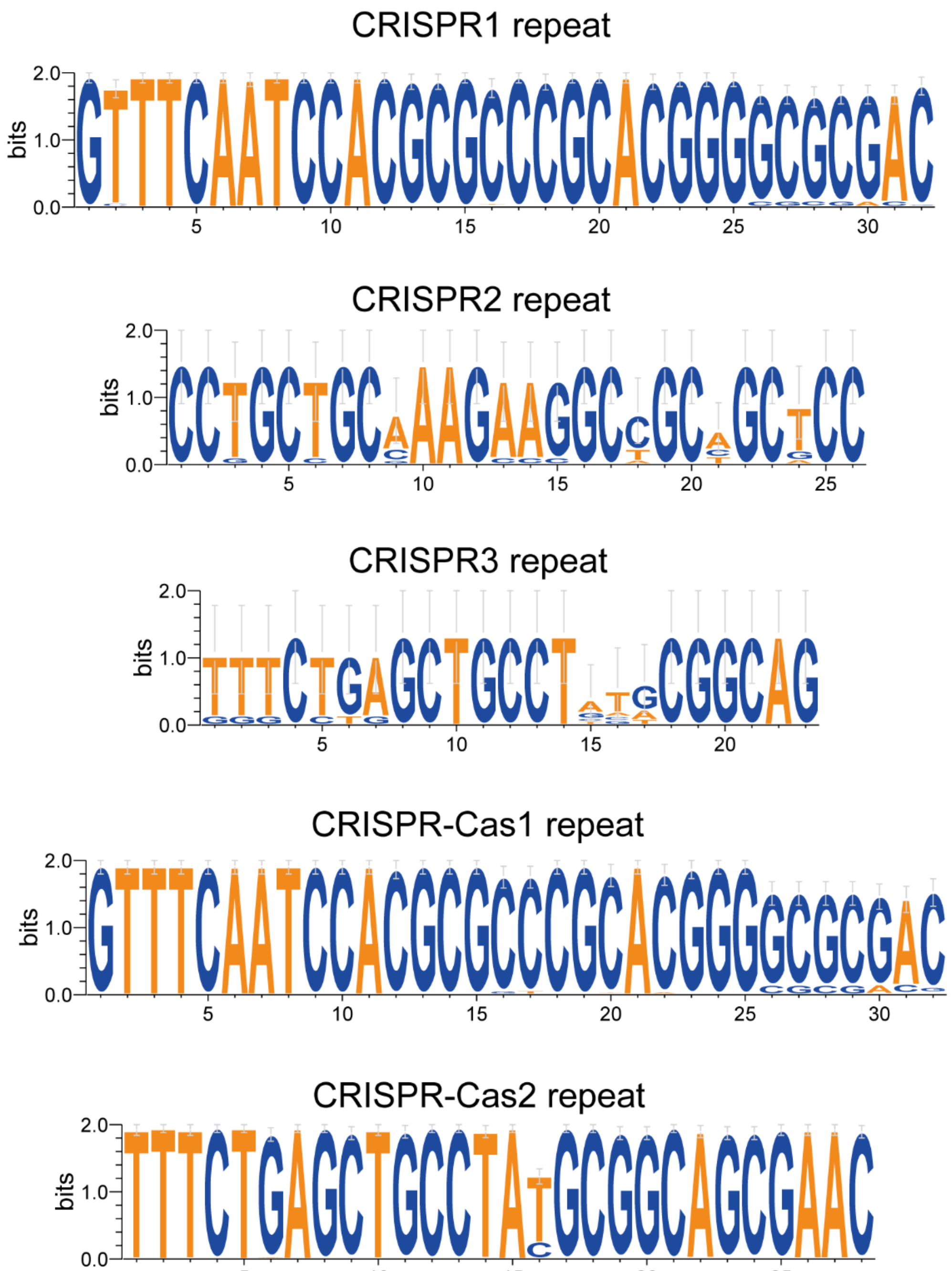

Figure S2. The sequence logo for the direct repeat (DR) sequences in each CRISPR locus.

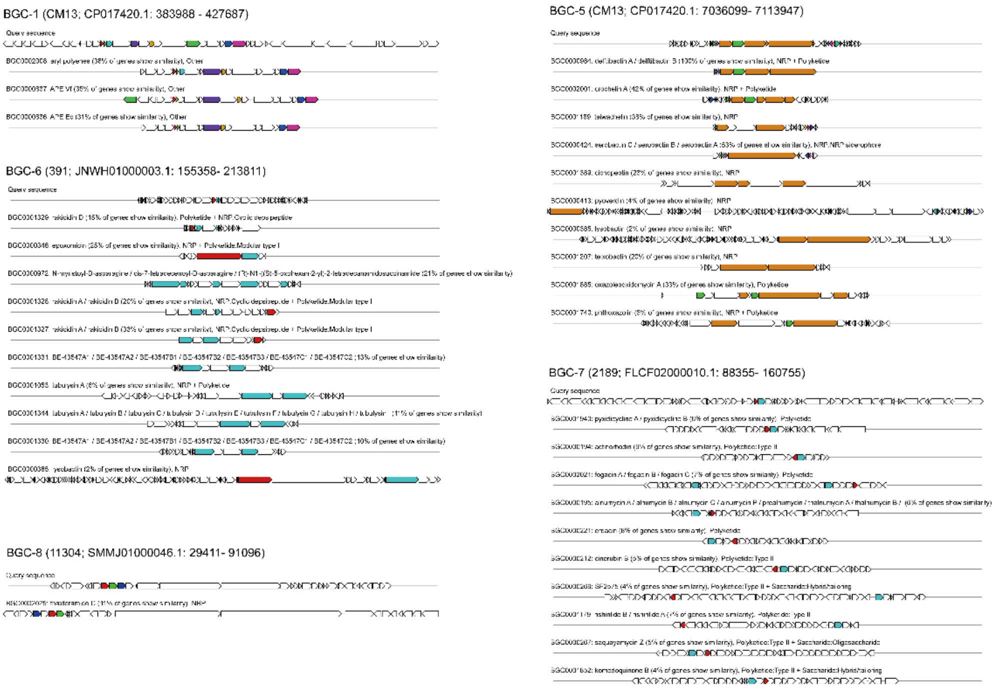

Figure S3. Comparative analysis of biosynthetic gene clusters for secondary metabolism from *D. tsuruhatensis* and other strains. Different genes are in different colors, and genes with the same color are homologous to each other.

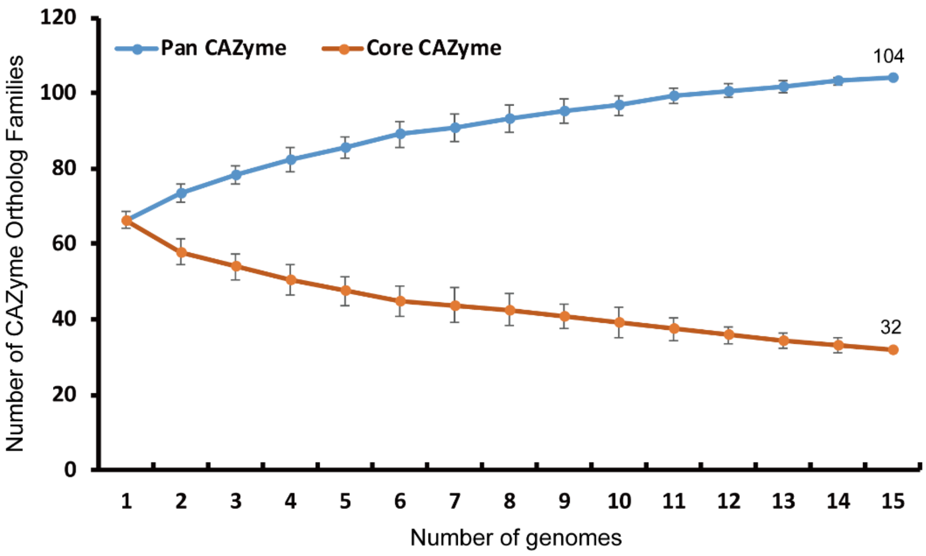

Figure S4. Core and pan-CAZymes curves show the downward trend of the core CAZyme families and the upward trend of the pan- CAZyme families with the increase in the number of genomes.
